# Spiral-Grating Tapered Gold Tip Used for Micro-Nanoscale Multi-Functional Sensing

**DOI:** 10.3390/s26020704

**Published:** 2026-01-21

**Authors:** Rongtao Huang, Yuxin Chen, Zhi-Yuan Li

**Affiliations:** 1 School of Physics and Optoelectronics, South China University of Technology, Guangzhou 510641, China; 2 State Key Laboratory of Luminescent Materials and Devices, South China University of Technology, Guangzhou 510640, China

**Keywords:** spiral-grating tapered gold tip, SPR sensing, plasmonic enhanced fluorescence, scanning near-field optical microscopy

## Abstract

Optical fiber surface plasmon resonance (SPR) sensing, as a label-free, highly sensitive, rapid-response and in situ detection technology, has demonstrated significant utility in various physical, chemical and biological detection applications. This paper focuses on a fiber-integrated microscale spiral-grating tapered gold tip SPR sensor. We first introduce the working principle and sensing capability with high space–time resolution of this SPR microsensor. Then we provide a comprehensive description of its application in the study on the important fundamental scientific issue of liquid–liquid diffusion. Finally, we demonstrate the application of the spiral-grating tapered gold tip to plasmonic enhanced fluorescence and scanning near-field optical microscopy. By systematically summarizing the excellent multifunctional sensing performance of the microscale spiral-grating tapered gold tip, this paper aims to provide new optical schemes and tools for the study on complex physicochemical processes and light-matter interactions at microscale and nanoscale.

## 1. Introduction

With the development of society and the improvement in life quality, there is an increasingly high demand for biochemical testing at present. Among various sensing technologies, surface plasmon resonance (SPR) sensing technology, as a highly sensitive, fast-response and label-free sensing technology, has developed vigorously in recent years [1,2,3,4,5,6]. Compared with the traditional Kretschmann prism SPR sensor, the optical fiber SPR sensors that combine SPR sensing with optical fiber not only retain the inherent advantages of SPR sensing, but also offer the significant advantages of miniaturization, flexible operation and in situ measurement [6,7,8,9,10,11]. For optical fiber SPR sensors, the most crucial aspect is to design a specific structure that enable the light transmitted in the fiber core to be incident on the metal structure, thereby exciting surface plasmon polariton (SPP) or local surface plasmon resonance (LSPR). For a particular structure, the SPR mode can be excited with the illumination of light at the wavelength that satisfies the resonance condition, such as the momentum matching condition for the excitation of SPP. A change in the refractive index (RI) of the medium on the metal surface will alter the resonance condition, leading to a sensitive shift in the resonance wavelength, i.e., the SPR wavelength. Based on the sensitive response of the SPR wavelength to the change in RI, the RI can be measured by detect the SPR wavelength.

**Figure 1:**
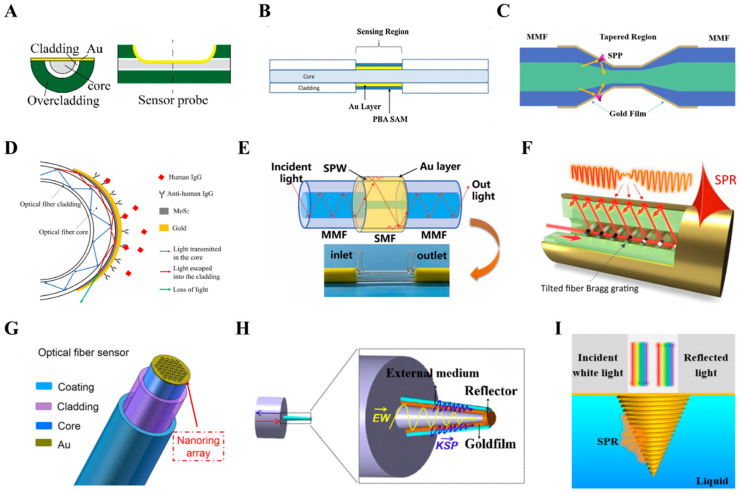
Structures of optical fiber SPR sensors. (**A**) D-shaped fiber [12]. (**B**) Unclad fiber [19]. (**C**) Tapered fiber [26]. (**D**) U-shaped fiber [33]. (**E**) Hetero-core fiber [38]. (**F**) Fiber grating [44]. (**G**) Nanoaperture array structure [45]. (**H**) Micro tapered tip [48]. (**I**) Spiral-grating tapered gold tip [54].

It is an effective method to destroy the optical fiber cladding by polishing or grinding to leak out the light transmitted in the fiber core. In this method, part or all of the fiber cladding is removed from a small section of the fiber and coated with a metal film to form a measuring region, so that the light transmitted in the fiber core is directly coupled to the metal film on the surface of the measuring region to excite SPR for sensing. When the RI of the medium on the surface of the metal film changes, the resonance condition changes accordingly, resulting in the SPR wavelength shift, based on which high sensitivity detection is achieved. In order to improve the sensing performance, optimizing the polishing length, controlling the thickness of the metal film, and introducing other metal nanostructures (e.g., metal gratings, metal nanoparticles) to enhance the local field effect are often used. This kind of fiber structure mainly includes the D-shaped fiber [12,13,14,15,16,17] and the unclad fiber [18,19,20,21,22,23]. The sizes of these sensors mainly range from 5 mm to 20 mm, and they have shown good application prospects in the fields of chemistry, biology, medicine and so on. Cao et al. fabricated a 5 mm-long gold-coated D-shaped fiber SPR sensor at a low refractive index polymer fiber, as shown in Figure 1A [12], which showed excellent performance of 24.50 nm/wt% in glucose solution detection. Qian et al. achieved high sensitivity for glycoprotein detection by fabricating a gold-coated unclad fiber SPR sensor on a multimode fiber (Figure 1B) and functionalizing it with phenylboric acid [19].

Another method is to change the structure of the optical fiber without damaging the cladding structure, so that the light transmitted in the core enters the cladding and SPR is excited on the metal film outside the cladding to achieve sensing. Such structures include tapered fiber [24,25,26,27], U-shaped fiber [28,29,30,31,32,33,34,35,36], hetero-core fiber [37,38], fiber grating [39,40,41,42,43,44], etc. The sizes of these sensors are predominantly on the order of 10 mm. As shown in Figure 1C,D [26,33], the taper fiber and U-shaped fiber structures allow the fiber core light field to leak out into the fiber cladding, thereby enhancing the interaction between the light and the metal film on the cladding surface. As shown in Figure 1E, the hetero-core fiber structure utilizes the mode field mismatch between different optical fibers, such as between single-mode fiber (SMF) and multi-mode fiber (MMF), to allow the light transmitted in the core to enter the cladding, thereby exciting SPR on the metal film on the surface of the cladding [38]. As shown in Figure 1F [44], by etching a fiber grating structure into the fiber core, the effective coupling of optical wave energy from the fiber core to the fiber cladding can be achieved without changing the original structure of the fiber. This enables high-efficiency and high-stability fiber SPR sensing. Wang et al. achieved in situ, real-time, and quantitative monitoring of ion dynamics at the electrode interface in a working battery by using a tilted fiber Bragg grating (TFBG)-SPR sensor [44], demonstrating the highly sensitive response capability of such a sensor in a complex electrochemical environment.

Exciting SPR of the metal microstructures on the optical fiber end face through the emitted light from the fiber core is a method that does not require any processing of the fiber structure. In this scheme, the role of the fiber is to conduct the excitation light and collect the reflected light signal. The structures of these sensors are mainly divided into two categories. One is the etching nanoaperture array structure on the metal film based on the optical fiber end face [45,46,47], as shown in Figure 1G [45]. For this structure, the sensing function is realized by the LSPR effect of nanoapertures, which is significantly enhanced by the accompanying SPP and diffractive modes arising from the periodic array. This structure has a high level of integration with a longitudinal size of tens of nm and a transverse size of ~100 μm and can be used to achieve RI detection with high sensitivity and high stability. In addition to etching, fabricating a specific structure substrate on the fiber end face and plating it with a metal film is also a common scheme for fabricating micro-tip SPR sensors [48,49,50,51]. As shown in Figure 1H, Kong et al. prepared a micro tapered structure SiO_2_ substrate on the fiber end face by controlling the discharge parameters of the fiber welding machine and plated a gold film on it to fabricate a tapered plasmonic microsensor with a volume size on the order of 10 × 10 × 10 μm^3^ [48]. In principle, this micro tapered structure can be viewed as a Kretschmann prism coupling structure. With the irradiation of the transmitted light of the fiber core, SPP can be excited on the surface of the gold film to achieve the highly sensitive refractive index detection.

Based on the above schemes, various optical fiber SPR sensors have been vigorously developed and manufactured in recent years and widely used in biomedical detection, new energy engineering and other fields, showing broad application prospects. Among them, the micro-tip sensors fabricated on the fiber end face show great potential due to their small size and can be applied to the detection of a single cell level. In recent years, the development of the direct laser writing (DLW) method based on the two-photon polymerization (2PP) technology [52,53] has provided great support for such a scheme, by which microsensors with diverse functions can be designed. The fiber-integrated spiral-grating tapered gold tip shown in Figure 1I [54] is a typical representative, which can be applied not only as an SPR sensor with high space-time resolution, but also for fluorescence detection and near-field optical imaging with excellent performance. This paper systematically introduces the principle of the spiral-grating tapered gold tip and its multifunctional sensing applications, aiming to provide new experimental schemes and tools for research in the fields of physics, chemistry, biomedicine and material science.

## 2. Sensing Principle of the Spiral-Grating Tapered Gold Tip

**Figure 2:**
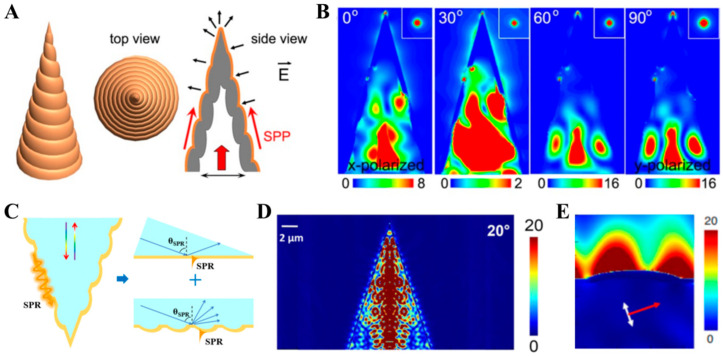
The hollow and solid spiral-grating tapered gold tips. (**A**,**B**) The hollow spiral-grating tapered gold tip [55]. (**A**) A schematic diagram of the working principle. (**B**) Electromagnetic field calculation results. (**C**–**E**) The solid spiral-grating tapered gold tip. (**C**) A schematic diagram of the working principle [54]. (**D**,**E**) Electromagnetic field calculation results [56,57].

In 2013, Li et al. proposed and fabricated a hollow spiral-grating tapered gold tip structure based on the end face of the optical fiber (shown in Figure 2A) [55]. Viewed along each generatrix, such a structure can, in principle, be equivalent to a grating coupled structure. SPP can be excited on the gold film surface with the irradiation of the light transmitted in the fiber core. As shown in Figure 2B, for this hollow spiral-grating tapered gold tip, the excitation and focusing of the SPP can be achieved with the irradiation of incident light at any polarization state because the spatial symmetry of the structure is destroyed by the spiral grating. Due to the asymmetry of the spiral grating, the SPP undergoes constructive interference at the apex of the hollow spiral-grating tapered gold tip, thereby forming a deep sub-wavelength near-field bright light spot. In contrast, the solid spiral-grating tapered gold tip as shown in Figure 2C, which was developed based on the hollow gold tip, ingeniously combines the Kretschmann prism coupling structure and the grating coupling structure [54,56,57]. Under the coordinated effect of the dual coupling mechanism, the excitation efficiency of SPP is significantly improved. For the Kretschmann prism structure, the excitation of SPP requires that the incident light meets the momentum matching condition, which is as follows:

(1) $$k_{SPP}=k_{0}{\left(\frac{\varepsilon_{d}\varepsilon_{m}}{\varepsilon_{d}+\varepsilon_{m}}\right)}^{\frac{1}{2}}=k_{inc}=\frac{\omega }{c}\sqrt{\varepsilon_{p}}sin\left(\theta_{SPP}\right)$$

where *k*_0_ *= 2π/λ_SPP_*, *ω* is the angular frequency of incident light, and *c* is the light speed in vacuum. *λ_SPP_* is the surface plasmon resonance (SPR) wavelength for exciting SPP. *ε_p_*, *ε_m_* and *ε_d_* are the permittivity of the structure internal substrate, the gold film and the external medium. Combined with the grating coupling structure, since gratings can provide momentum compensation for the excitation of SPP, the momentum matching condition is as follows:

(2) $$k_{SPP}=k_{0}{\left(\frac{\varepsilon_{d}\varepsilon_{m}}{\varepsilon_{d}+\varepsilon_{m}}\right)}^{\frac{1}{2}}=k_{inc}+qK=\frac{\omega }{c}\sqrt{\varepsilon_{p}}sin\left(\theta_{SPP}\right)+qK,\;q=0,\pm 1,\pm 2\cdots \;$$

where *q* is the diffraction order and *K = 2π/Λ* is the grating reciprocal vector.

According to this equation, when the refractive index of the external medium changes, the momentum matching condition will also change, causing the SPR wavelength that meets the matching condition to change. Therefore, by detecting the SPR wavelength, the refractive index can be measured, and this is the SPR sensing principle. In addition, Equation (2) indicates that the combination of the grating structure can make the structural design of the gold-tip sensor more flexible, enabling it to meet the requirements of various applications. The electromagnetic field calculation results shown in Figure 2D,E [56,57] both indicate that such a structure can effectively excite SPP and form a “hot spot” at its apex with the irradiation of incident light. In this paper, the spiral-grating tapered gold tip refers specifically to the solid spiral-grating tapered gold tip.

## 3. Spiral-Grating Tapered Gold Tip Used for SPR Sensing

**Figure 3:**
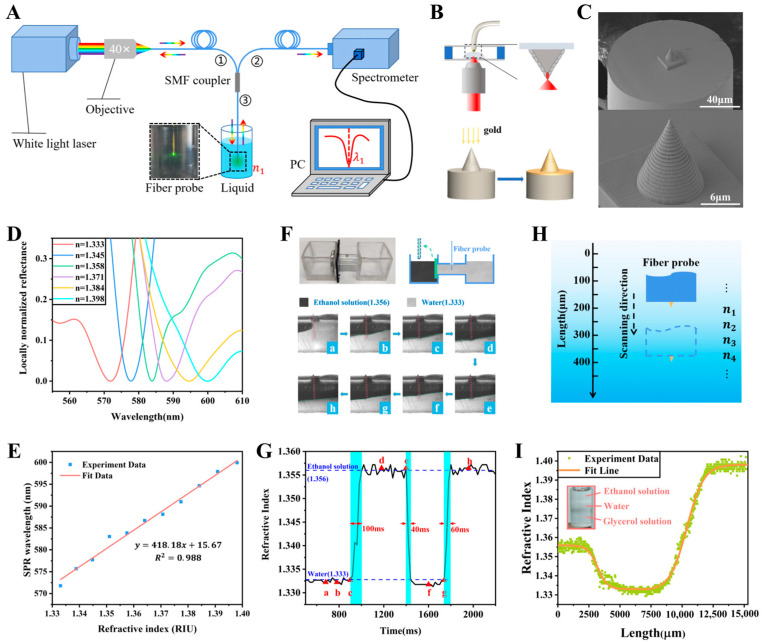
Spiral-grating tapered gold tip used for SPR sensing. (**A**) A schematic diagram of the experimental setup [58]. (**B**) A schematic diagram of the two-step preparation process of the gold tip [58]. (**C**) Scanning electron microscope (SEM) images of the experimentally fabricated gold tips [58]. (**D**,**E**) RI sensitivity of the gold-tip microsensor [59]. (**F**,**G**) Monitoring of the rapid RI change process with the gold-tip microsensor. (a)–(h) represent the different stages of convection [58]. (**H**,**I**) Measurement of the gradient RI distribution with the gold-tip microsensor [58].

Based on the SPP excited on the surface of the spiral-grating tapered gold tip, the spiral-grating tapered gold tip was applied as a fiber-integrated SPR microsensor for detecting the RI of liquids [58,59]. The experimental setup is shown in Figure 3A [58]. With the illuminating of white light transmitted in the optical fiber, the light of SPR wavelength can excite SPP on the surface of the gold-tip microsensor, while the light of other wavelengths is reflected back into the optical fiber and collected by a spectrometer. The SPR wavelength can be obtained by analyzing the reflection spectrum. Experimentally, the spiral-grating tapered gold tip was fabricated by the two-step method (Figure 3B): first, a spiral-grating tapered polymer substrate is fabricated on the end face of optical fiber by the DLW method based on the 2PP technology; and then a ~90 nm thick gold thin film is conformally coated on the surface of polymer substrate by the magnetron sputtering method [58]. Figure 3C shows the experimentally fabricated gold tips with a relatively smooth surface, a bottom diameter of ~10 μm, and a height of ~14 μm [58]. Along the generatrix direction, the period of the spiral grating is ~700 nm. The reflectance spectra of the spiral-grating tapered gold tip measured in liquids with different RIs, as shown in Figure 3D [59], indicate that the gold-tip microsensor can achieve a RI sensitivity of 418 nm/RIU (Figure 3E). Importantly, thanks to the fast SPP response and small structure, the gold-tip microsensor can be used to detect RI with a fast time response of ~20 ms (limited by the integration time of the spectrometer) and a high space resolution better than 14 μm. The RI sensitivity of 418 nm/RIU is close to that of widely used TFBG-SPR sensors (~500 nm/RIU [39,40,41,42]) and fiber-integrated nanoaperture array SPR sensors (~500 nm/RIU [45]) and is thus sufficient to meet the demands of most physical and biochemical detection, indicating that the gold-tip microsensor can achieve both favorable RI sensitivity and high space resolution.

To demonstrate such capabilities, the gold-tip microsensor was used to measure fast RI change processes and gradient RI distributions in liquids [58]. As shown in Figure 3F, the microsensor was placed in a pipe with the convection of ethanol solution and water, and the fast RI change process was monitored as shown in Figure 3G. It can be seen in the curve that the microsensor can be used to measure the RI change process for tens of milliseconds. The microsensor was used to scan liquids and detect RI point by point to measure the RI space distributions, as shown in Figure 3H. Figure 3I shows the RI distribution curve measured by the microsensor scanning the ethanol solution–water–glycerol solution layered liquid. It can be seen that the microsensor can be used to accurately measure the RI distribution caused by the gradient distribution of components in the two boundary layers of the liquid. The RI measurement of liquids with a high space–time resolution indicates that the spiral-grating tapered gold tip microsensor can serve as a key component in constructing sensing systems for monitoring dynamic space–time processes in liquids, including important liquid–liquid diffusion processes.

## 4. In Situ Monitoring of Liquid–Liquid Diffusion

### 4.1. Conventional Optical Methods for Monitoring Liquid–Liquid Diffusion

Diffusion, a mass transfer process that widely exists in nature and is closely related to our daily lives, is significantly importance in the fields of physics, chemistry and biology. Diffusion phenomena had already attracted attention and been studied even before the 19th century [60]. In 1855, Adolf Fick proposed the well-known diffusion law to quantitatively describe diffusion processes [61], and introduced an important physical quantity, the diffusion coefficient. According to Fick’s law, once the diffusion coefficient of a system is determined, the diffusion process can be quantitatively calculated. Therefore, the diffusion coefficient has become the focus of the research on diffusion. However, the molecule distribution in liquids is neither as diluted as in gases nor as ordered as in solids, leading to non-negligible and complex intermolecular interactions. Consequently, the liquid–liquid diffusion systems are highly complex [62,63]. Up to now, there is no perfect theory for them, and current research on them still relies predominantly on experimental measurements. With the development of liquid–liquid diffusion measurement experiments for many years, a variety of experimental methods for measuring liquid–liquid diffusion coefficients have been developed, including non-optical methods (e.g., diaphragm cell method [64,65,66,67,68,69,70], the Taylor dispersion method [71,72,73,74,75,76]) and optical measurement methods. Among them, the optical measurement methods can not only measure the diffusion coefficient but also monitor the concentration space–time evolution during the diffusion process, which are more perfect for the research on diffusion process.

**Figure 4:**
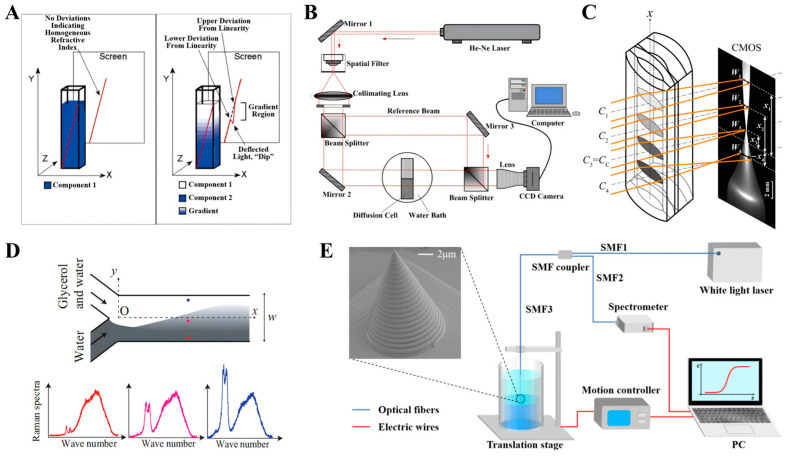
Optical methods for monitoring liquid–liquid diffusion processes. (**A**) The light beam deflection method [78]. (**B**) The interferometry method [84]. (**C**) The liquid-core cylindrical lens method [98]. (**D**) The microfluidic method [105]. (**E**) The spiral-grating tapered gold tip microsensor scanning method [54].

The light beam deflection method was initially proposed by Wiener in 1893 [77]. The principle of this method is that light is deflected as it passes through an inhomogeneous medium, as shown in Figure 4A [78]. By measuring the deflection angles at different positions, the RI distribution can be calculated to obtain the concentration distribution. The diffusion process is analyzed based on the concentration distribution at different times. This method is simple to operate and has been applied to measure diffusion coefficient for various liquid systems [78,79,80,81,82,83]. However, its measurement accuracy and space resolution are poor due to the large width of the beam. The interference method is to record the interference fringes and then restore the phase information by numerical calculation to obtain the concentration distribution. Its basic principle is that the optical path length varies when light passes through liquids with different RIs, resulting in a phase difference. The commonly used experimental device is shown in Figure 4B [84]. This method is considered to be a highly accurate method, so studies utilizing it to measure liquid–liquid diffusion coefficients by it have been extensively reported [84,85,86,87,88,89,90,91,92,93,94]. However, it requires extremely high stability in experimental operation, and the recorded information is global, which causes the measurement accuracy of local concentration changing depending on the recovery algorithm. The liquid-core cylindrical lens method is to inject the diffusing liquid into a specially structured hollow cylindrical lens, as shown in Figure 4C, and measure different RI based on the principle of different RIs forming different focal lengths to obtain the concentration distribution [95,96,97,98]. This method has the advantages of simple requirement for experimental environment, fast measurement and intuitive experimental results. The primary shortcoming of this method is that the spherical aberration of the measurement system restricts the accurately measured RI range in a small range, limiting its widespread application. For all of the above methods, the measurement results are actually the overall contribution of the transverse RIs because the measurement light passes consecutively through the container wall and the liquid, so their accuracy is less reliable because of the inevitable disturbance by the vessel wall, liquid level bending, and so on.

With the development of manufacturing technology, microfluidic tubes have been applied to research liquid–liquid diffusion in recent years [99,100,101,102,103,104,105,106,107,108,109]. Among them, the Y-shaped microfluidic tube is the most characteristic [102,103,104,105,106,107,108,109]. As shown in Figure 4D [105], the two liquids are injected into the two branches of the Y-shaped tube to form a diffusion mixed flow in the main channel, and then the concentration distribution of the diffusion mixed flow area is measured by fluorescence, infrared, or Raman imaging. The significant advantage of this method is that the time evolution of concentration is converted into space distribution, and the diffusion process is analyzed by the space distribution of concentration in the main channel, so it does not place stringent demands on the time response of the imaging technology. When using Raman imaging, the concentration distribution is obtained by measuring the Raman spectra point-by-point by focusing the laser beam, and the space resolution can reach micrometer order, which is superior to the above methods. However, due to the small space range for concentration measurement in the microchannel, this method cannot be used to monitor diffusion processes in a large space-time range. In addition, the influence of the two liquid flows on the diffusion process and the non-uniform flow rate in the same cross-section reduce the measurement precision.

### 4.2. The Spiral-Grating Tapered Gold Tip Microsensor Scanning Method

By comparing the above methods, it can be seen that these methods have their own characteristics, but they are unable to accurately measure the evolution of concentration distribution during the diffusion process in a large space–time range with high space resolution, which limits their measurement accuracy. Therefore, it is very important to develop new measurement methods to monitor the diffusion process in a large space–time range with high space resolution. In order to solve this problem, a new measurement scheme is recently proposed to in situ measure the concentration space–time diagram for the diffusion process by the spiral-grating tapered gold tip microsensor [54,59]. This method is based on the one-to-one relationship between the RI and the concentration of binary mixtures, such as different concentrations of glycerol aqueous solutions have different RIs. The experimental setup is shown in Figure 4E [54]. By using a stage to control the microsensor to repeatedly scan the diffusing liquid, the space distribution of concentration at different times can be measured to obtain the concentration space–time diagram. Due to measurements with a larger space–time range and a high space resolution, this method can be used to in situ monitor the diffusion processes with high accuracy and reveal the diffusion laws.

### 4.3. In Situ Monitoring of Non-Fickian Liquid–Liquid Diffusion

**Figure 5:**
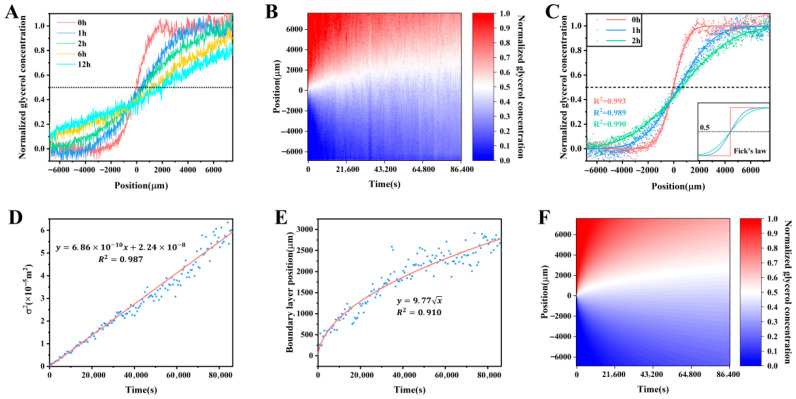
The glycerol concentration space–time evolution for the water–50% glycerol solution diffusion system [59]. (**A**,**B**) Glycerol concentration distribution data measured by the gold-tip microsensor scanning method. (**C**–**E**) Fitting of the experimental data by the non-Fickian diffusion model. (**F**) Space–time diagram of glycerol concentration calculated by the non-Fickian diffusion model.

Based on the spiral-grating tapered gold tip microsensor scanning method, the water–glycerol diffusion system [59], water–sodium chloride diffusion system [54] and ethanol solution–water system [110] have been studied. Figure 5A shows the glycerol concentration space distribution curves at the time of 0, 1, 2, 6, and 12 h measured by scanning the microsensor in the water–50% glycerol solution diffusion liquid. Based on the space distribution of glycerol concentration at different time, the space–time diagram of glycerol concentration as shown in Figure 5B was obtained. Its spatial range is ~14.3 mm with a space solution of ~15.2 μm and time range is 24 h with a time solution of 10 min. Such large space–time range and high space resolution allow this concentration space–time diagram to accurately describe the diffusion process. Both the concentration distribution curves and the concentration space–time diagram show that the center of the diffusion layer moves to the glycerol solution side during the water–50% glycerol solution diffusion process. This overall directional movement phenomenon of the diffusion layer is thought to be caused by the obstruction caused by the intermolecular interaction forces during the diffusion process. As a complete unit, the surface tension of water is higher than that of the glycerol solution, and this tension difference causes a barrier in the diffusion layer. When glycerol molecules diffuse towards the water side, they need more energy and time to overcome this barrier. Numerically, the new non-Fickian diffusion model is constructed by introducing an equivalent surface tension effect into the Fickian diffusion model, which is as follows:

(3) $$C\left(z,t\right)=\frac{C_{1}+C_{2}}{2}+\frac{C_{1}-C_{2}}{2}erf\left(\frac{z-A\sqrt{t}}{2\sigma \left(t\right)}\right)$$

Here, *A* is a proportionality constant related to the magnitude of the equivalent surface tension, and $A\sqrt{t}$ is used to describe the amount of diffusion layer movement. $\sigma \left(t\right)\;=\sqrt{Dt}$ represents the width of the diffusion layer. In this model, the overall liquid–liquid diffusion behavior manifests as a non-Fickian diffusion layer center position shift with the movement amount proportional to $\sqrt{t}$ superimposed on a Fickian diffusion layer broadening with the diffusion coefficient of *D*. As shown in Figure 5C–E, this model was used to fit the experimental data well, and the diffusion coefficient for the water–50% glycerol solution system was obtained as 6.86 × 10^−10^ m^2^/s. Combining the fitting data shown in Figure 5D,E and Equation (3), the concentration space–time diagram shown in Figure 5F was calculated, which is basically consistent with the experimentally measured concentration space–time diagram shown in Figure 5B, indicating that the non-Fickian model can accurately explain the water–50% glycerol solution diffusion process [59].

**Figure 6:**
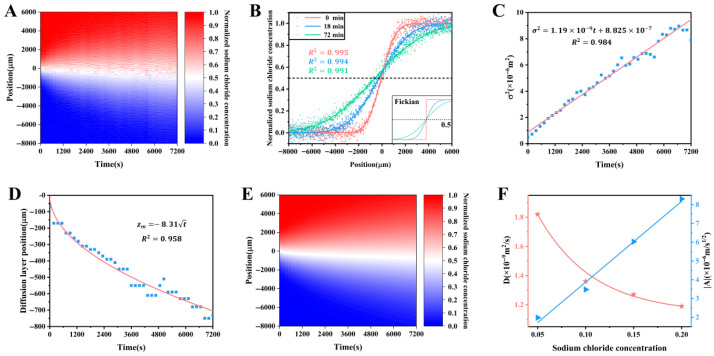
The water–sodium chloride solution diffusion system [54]. (**A**) The space–time diagram of sodium chloride concentration during the water–20% sodium chloride solution diffusion process. (**B**–**E**) Fitting of the experimental data for the water–20% sodium chloride solution diffusion system by the non-Fickian diffusion model. (**F**) Relationships of the diffusion coefficient and the magnitude of the constant *A* versus the concentration of sodium chloride solution.

The microsensor was used to scan the diffusion liquid of water–20% sodium chloride solution repeatedly, and the space–time diagram of sodium chloride concentration during the diffusion process was monitored, as shown in Figure 6A. Its space range is ~14 mm with a space resolution of ~15.3 μm, and the time range is 2 h with a time interval of 3 min. It shows that the diffusion layer center of the one-dimensional water–20% sodium chloride solution diffusion system moves to the water side during the diffusion process. The overall directional movement of the diffusion layer is also explained by the tension difference between the upper and lower interfaces of the diffusion layer. As a complete unit, the surface tension of the sodium chloride solution is higher than that of water, and this tension difference causes a potential barrier in the diffusion layer. As the water molecules move to the sodium chloride solution side through the diffusion layer, they take more energy and time. Numerically, the concentration data were fitted by the non-Fickian diffusion model (Equation (3)). As shown in Figure 6B–D, this diffusion model was used to fit the experimental data well, and the diffusion coefficient for this system was obtained as 1.19 × 10^−9^ m^2^/s. Combining the fitting data and Equation (3), the concentration space–time diagram was calculated as shown in Figure 6E. Comparing Figure 6A,E, it can be seen that the two diagrams are basically consistent, indicating that this non-Fickian model can accurately explain the water–20% sodium chloride solution diffusion process. In order to further explore the diffusion law, the diffusion processes of water and 15%, 10% and 5% sodium chloride solution were measured by the same method, and the relationships of the diffusion coefficient and the proportionality constant *A* versus the concentration of sodium chloride solution were obtained by data fitting, as shown in Figure 6F. It shows that for the diffusion system of water and the sodium chloride solution with higher concentration, the diffusion coefficient is smaller, and the diffusion layer movement account is great. The absolute value of the constant *A* shows a positive linear relationship with the sodium chloride concentration, which is consistent with the linear relationship between the surface tension and the concentration for the sodium chloride solution, which further shows the accuracy of the non-Fickian diffusion model constructed by introducing the equivalent surface tension [54].

**Figure 7:**
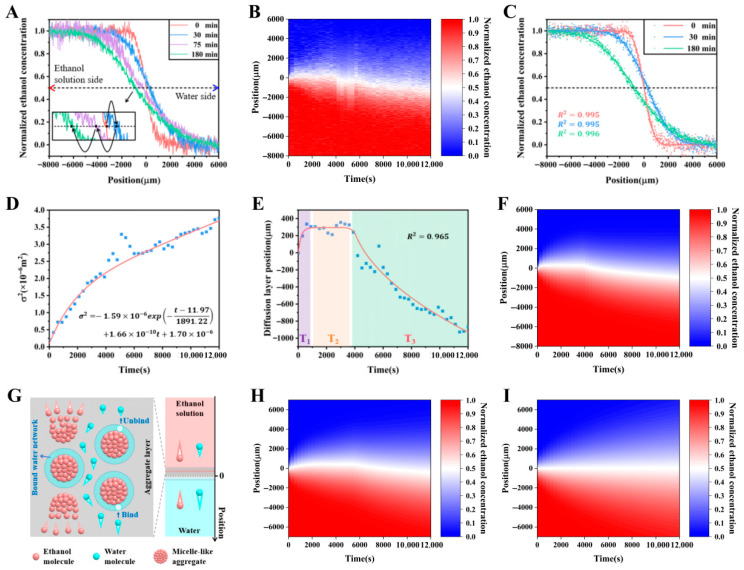
The ethanol solution-water diffusion system [110]. (**A**–**F**) The 50% ethanol solution–water diffusion process. (**A**,**B**) Ethanol concentration distribution data measured by the gold-tip microsensor scanning method. (**C**–**F**) Fitting of the experimental data. (**G**) Effect of molecular self-assembly behavior on the ethanol solution-water diffusion process. (**H**) The 40% ethanol solution–water diffusion process. (**I**) The 30% ethanol solution–water diffusion process.

The microsensor was used to scan the 50% ethanol solution-water diffusion liquid repeatedly and the time evolution of the ethanol concentration distribution was measured, as shown in Figure 7A. According to the ethanol concentration distribution at different times, the space–time diagram of ethanol concentration as shown in Figure 7B was obtained, whose space range is ~14 mm with a space resolution of ~15.3 μm, and time range is 200 min with a time resolution of 5 min. It shows that the diffusion layer center moves in a three-stage manner during the diffusion process, first moving to the water side, then staying constant for a period of time, and finally moving to the ethanol solution side. In order to explore the diffusion law, the Fickian diffusion model was modified to obtain the following non-Fickian diffusion model:

(4) $$C\left(z,t\right)=\frac{C_{1}+C_{2}}{2}+\frac{C_{1}-C_{2}}{2}erf\left(\frac{z-z_{m}\left(t\right)}{2\sigma \left(t\right)}\right)$$

where *z_m_*(*t*) represents the diffusion layer center position and *σ*(*t*) represents the diffusion layer width. As shown in Figure 7C, Equation (4) was used to well fit the experimental data to obtain *z_m_* and *σ* values at different times, which were used to analyze the effective diffusion coefficient and the diffusion layer center position during the diffusion process. The *σ*^2^-*t* relationship shown in Figure 7D indicates that the diffusion coefficient gradually decreases to a constant as diffusion proceeds, and the *z_m_*-*t* relation shown in Figure 7E demonstrates that the diffusion layer center exhibits a three-stage movement. Combining Equation (4) and the fitting data in Figure 7D,E, the fitted space–time diagram of ethanol concentration was obtained, as shown in Figure 7F, which is basically consistent with the experimental space–time diagram shown in Figure 7B. Neither the decay of the diffusion coefficient nor the movement of the diffusion layer follows the Fick’s law, indicating that the 50% ethanol solution–water diffusion process seriously deviates from the Fickian diffusion model. To explain the exotic diffusion behavior, the diffusion process was observed in the experiment to be accompanied by the molecular self-assembly phenomenon caused by hydrophobic interaction. As shown in Figure 7G, this molecular self-assembly behavior hinders the diffusion of ethanol molecules and water molecules. The decay of the diffusion coefficient and the three-stage movement of the diffusion layer are well-explained by combining the molecular self-assembly behavior, the excess volume effect, and the interfacial tension effect. Further experiments show that the 40% ethanol solution–water system, which is also accompanied by molecular self-assembly phenomenon, exhibits similar diffusion behavior to the 50% system, as shown in Figure 7H. While the 30% ethanol solution–water system, without the accompanying molecular self-assembly phenomenon, exhibits Fickian diffusion behavior, as shown in Figure 7I. These further confirm the key role of molecular self-assembly in the exotic diffusion process [110].

The non-Fickian diffusion phenomena observed in water–glycerol solution, water–sodium chloride solution, and ethanol solution–water systems indicate that the non-negligible interaction in liquid can significantly affect the macroscopic diffusion process and illustrates the importance of more accurate monitoring of liquid–liquid diffusion process to reveal new diffusion laws. The microsensor scanning method combines the advantages of simple working principle, simple operation performance, clear measurement results, large monitoring spatial and temporal range and high spatial resolution, which cannot be achieved by other measurement methods. This fully demonstrates the practicability and powerful ability of the spiral-grating tapered gold tip microsensor with high space–time resolution in the study on liquid–liquid diffusion. It is expected to be applied to the study on mass transfer processes and other physicochemical processes in important systems.

## 5. Spiral-Grating Tapered Gold Tip Used for Other Sensing Detection

Currently, most fiber optic sensors exhibit versatile applications. The tapered fiber and U-shaped fiber, as the commonly used sensor structures for refractive index detection, can also be applied to fluorescence detection [111,112]. The compatible ability of fluorescence and label-free detection makes them flexible and powerful tools for biosensing technology, which is expected to play an important role in the medical, environmental and food industries. Francois et al. proposed a collection-mode SPR fiber sensor and successfully realized the synergistic application of SPR sensing and fluorescence sensing on the same device [113]. After the label-free SPR detection of influenza A virus, the specific fluorescence signal was excited and collected by introducing quantum dot labeling to complete the specific detection of the virus. This study demonstrates the advantages of multifunctional sensors for medical detection applications. For the spiral-grating tapered gold tip, in addition to being used to detect the refractive index, it can be used to achieve enhanced fluorescence detection based on SPP on the gold film surface and for near-field optical imaging based on the sub-10 nm light spot at its apex.

### 5.1. The Spiral-Grating Tapered Gold Tip Used for Plasmonic Enhanced Fluorescence

**Figure 8:**
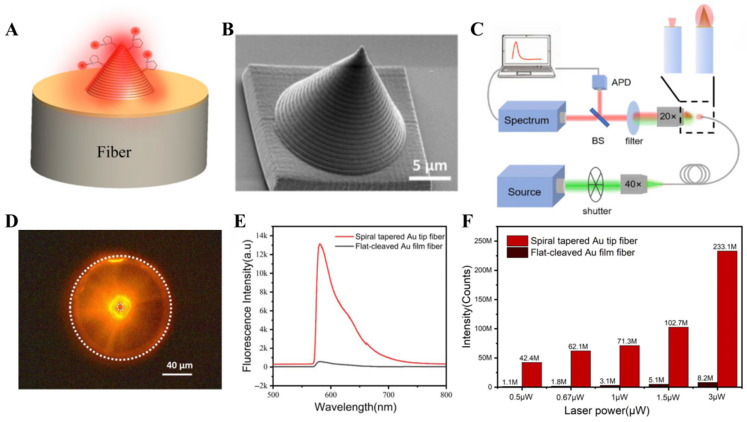
The spiral-grating tapered gold tip used for fluorescence detection [56]. (**A**) A schematic diagram of the principle of PEF achieved by the gold tip integrated on optical fiber end face. (**B**) SEM image of the experimentally fabricated gold tip. (**C**) Experimental setup for fluorescence detection. (**D**) CCD image of gold-tip excited fluorescence. (**E**,**F**) A comparison of performances between the gold-tip sample and the flat-cut gold film sample. (**E**) Fluorescence intensities. (**F**) Photon numbers.

In 2022, Long et al. applied the spiral-grating tapered gold tip as a probe to achieve plasmonic enhance fluorescence (PEF) [56]. As shown in Figure 8A, the incident light (532 nm) from the fiber efficiently excites surface plasmon polariton (SPP) on the gold tip surface, generating an extremely strong local electromagnetic field to substantially enhance the fluorescence of Rhodamine B (RhB) dye molecules adsorbed on the surface. The structure parameters of gold tip were optimized by simulation calculation, and then the spiral-grating tapered gold tip as shown in Figure 8B was fabricated on the end face of the fiber by the 2PP-based 3D printing technology and magnetron sputtering technology. Its bottom diameter is ~16 μm, height is ~13.8 μm and grating period is ~680 nm. As shown in Figure 8C, in the measuring fluorescence experiment, the beam generated by a 532 nm laser with a repetition rate of 80 MHz was introduced into the fiber to irradiate the sample. The emitted fluorescence was collected by a spectrometer to measure the spectrum and an avalanche photodiode (APD) to measure the photon numbers. The CCD image of the optical fiber end face shown in Figure 8D indicates that the RhB dye molecules on the surface of the spiral-grating tapered gold tip are excited to emit bright fluorescence with the illumination of incident light. In the experiment, the fluorescence intensity of RhB dye molecules on the gold-tip fiber end face was compared with that on the flat-cut gold film fiber. The fluorescence spectra shown in Figure 8E demonstrate that the fluorescence intensity of the gold-tip sample is about 23-fold higher than that of the flat-cut gold film sample with the illumination of light with same power (~3 μW). To verify the accuracy of the experiments, the total number of photons in the 0–12.5 ns time window of the two optical fibers at different incident intensities were tested for comparison (Figure 8F). The results show that the fluorescence intensity of the gold-tip sample is 38, 34.5, 23 and 20 times greater than that of the flat-cut gold film sample when the power of the incident light is 0.5, 0.67, 1 and 1.5 µW, respectively. These results indicate that the spiral-grating tapered gold tip can serve as an optical fiber PEF sensor to provide new means and methods for detection in biotechnology, clinical analysis and analytical chemistry.

### 5.2. The Spiral-Grating Tapered Gold Tip Used for Near-Field Optical Imaging

**Figure 9:**
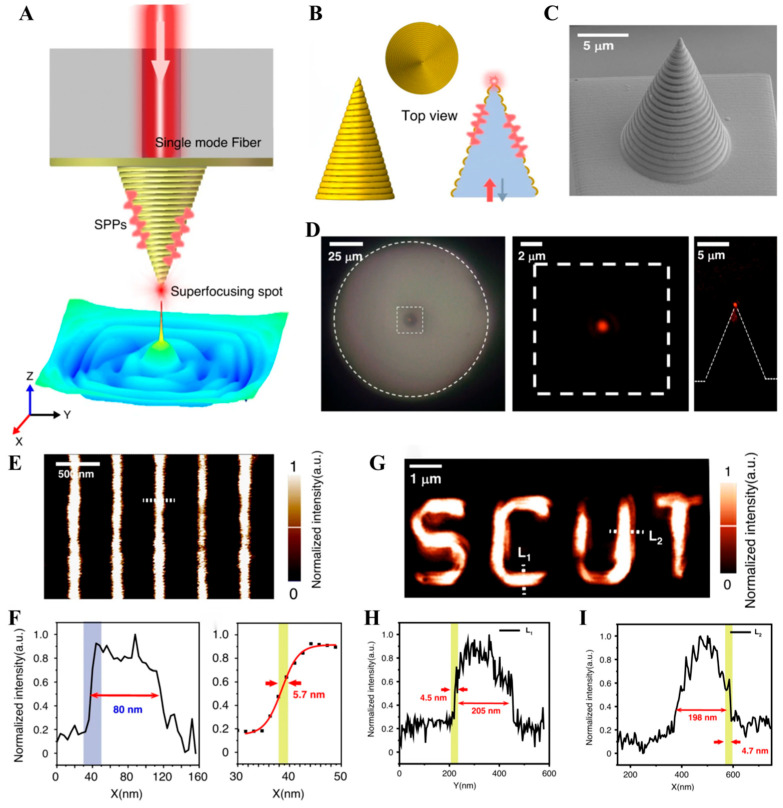
The spiral-grating tapered gold tip used for near-field optical imaging [57]. (**A**,**B**) A schematic diagram of the principle of the gold-tip SNOM probe. (**C**) SEM image of the experimentally fabricated gold-tip SNOM probe. (**D**) CCD image of light spot at the apex. (**E**,**F**) Scanning results of 1D periodic slits. (**G**–**I**) Scanning results of 2D “SCUT” pattern slits.

The numerical simulations indicate that, due to the disruption of spatial symmetry by the spiral grating, the spiral-grating tapered gold tip can achieve the excitation and focusing of SPP with the illumination of arbitrarily polarized incident light (linear polarized, circular polarized, vector polarized, etc.), causing a deep sub-wavelength superfocusing spot at the apex of the gold tip. This advantage allows the spiral-grating tapered gold tip to be applied as a near-field optical microscopy (SNOM) probe [57], as shown in Figure 9A,B. Figure 9C illustrates the SEM image of the gold tip at the fiber end face fabricated by the 2PP-based 3D printing technology and magnetron sputtering technology, and this gold tip can serve as a SNOM probe with the working wavelength of 785 nm. Its half conical angle is 20° with a bottom diameter of ~10.2 μm and a height of ~14 μm, and the grating period is ~750 nm. The experimental results show that the throughput of the 785 nm light irradiated from the fiber core into the spiral-grating tapered gold tip is close to 10%, and the light field distribution at the near field shows the output light is focused on the apex of the gold tip with a contrast of 20 dB, as shown in Figure 9D. The spiral-grating tapered gold tip was assembled in a commercial SNOM instrument (NTEGRA Solaris SNOM, NT-MDT), and the sample can be scanned based on the light spot the apex of the gold tip to study the optical properties of the sample. The scanning image of the chromium film standard sample consisted of one-dimensional (1D) periodic slits fabricated by focused ion beam (FIB) lithography is shown in Figure 9E. The light intensity curve corresponding to the dotted line in Figure 9E is shown in Figure 9F. The derivative result of the fitted curve of the blue area data indicates that the resolution of this gold-tip SNOM probe can reach approximately 5 nm, so the size of the light spot at the apex of the gold tip is about 5 nm, which is consistent with the theoretical calculation result. The scanning image of the chromium film engraved with the two-dimensional (2D) “SCUT” pattern slits is shown in Figure 9G. The light intensity curves corresponding to the two dashed lines in different directions in Figure 9G, shown in Figure 9H,I, also show the resolution of about 5 nm. The high-resolution imaging of 1D and 2D samples demonstrates that the spiral-grating tapered gold tip can serve as an excellent SNOM probe combined with the advantages of high resolution, high throughput, and high contrast.

**Figure 10:**
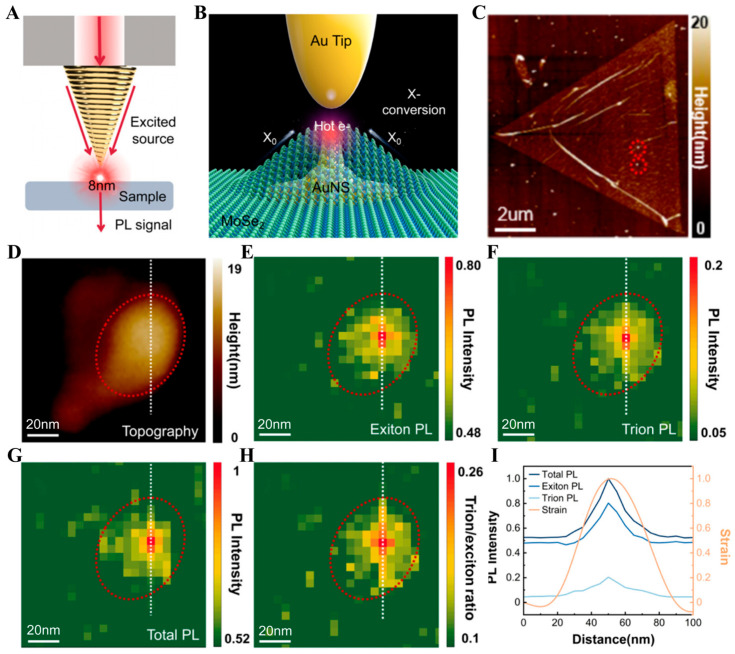
The spiral-grating tapered gold tip used for imaging of exciton PL and trion PL in the MoSe_2_/AuNS hybrid structure [114]. (**A**) Experimental setup. (**B**) A schematic diagram of the interaction between the gold tip and the hybrid structure. (**C**,**D**) AFM images of the MoSe_2_/AuNS hybrid structure. (**E**–**H**) PL intensity distribution of the MoSe_2_/AuNS hybrid structure. (**E**) Exciton PL. (**F**) Trion PL. (**G**) Total PL. (**H**) Ratio between the intensities of trion PL and exciton PL. (**I**) PL intensity and strain curves.

Based on the highly bright sub-10 nm light spot, the gold-tip SNOM probe can be used to investigate the optical properties of many important materials, including the photoluminescence (PL) of 2D materials. In 2025, Chen et al. achieved sub-10 nm visualization of exciton and trion in the MoSe_2_/gold nanostar (AuNS) hybrid structure by the spiral-grating tapered gold-tip SNOM probe with the working wavelength of 633 nm [114]. The half-conical angle of the 633 nm probe is 20°, consistent with that of the 785 nm probe, and the grating period is 620 nm. This indicates that the resonance wavelength of the gold tip can be tuned by adjusting the grating period, demonstrating the flexibility of the structure design. Figure 10A shows the schematic diagram of the SNOM detection system. The light of 633 nm is injected through the optical fiber to excite SPP on the surface of the gold-tip, forming an 8 nm light spot to irradiate the sample and excite PL. The PL signal generated by the sample is analyzed by combining the filter. Figure 10B illustrates the interaction between the gold-tip SNOM probe and the MoSe_2_/AuNS hybrid structure, including the exciton funnel effect induced by the MoSe_2_ strain caused by the AuNS and the injection of hot electrons to MoSe_2_. These hot electrons are generated by LSPR at the AuNS tip excited by the gold-tip SNOM probe. In the experiment, the MoSe_2_/AuNS hybrid structure was fabricated by covering a monolayer MoSe_2_ on the AuNS. The AFM images of this structure are shown in Figure 10C,D. The exciton PL, trion PL, and total PL intensity distributions of the MoSe_2_/AuNS hybrid structure were imaged with high space resolution using the gold-tip SNOM probe, as shown in Figure 10E–G. Figure 10H shows the distribution of the ratio of trion PL intensity to exciton PL intensity. Figure 10I shows the PL intensity curves and the strain distribution curve of the structure. They indicate that with the increase in strain, the emission intensity of excitons and trions increases significantly, and the proportion of trion PL also increases significantly. These results reveal that the synergistic mechanism of plasmon-induced hot electron injection and local strain-induced exciton funnel effect can significantly improve the efficiency of exciton-to-trion conversion at low strain.

## 6. Conclusions

In this paper, we have systematically reviewed the principle and applications of the multi-sensing functional spiral-grating tapered gold tip, aiming to provide new research schemes and practical experimental tools for studies in the fields of physics, chemistry and materials science. To begin with, the working principle of the spiral-grating tapered gold tip is introduced. It cleverly combines the prism structure and grating structure and can achieve the excitation and focusing of the SPP with the irradiation of the light transmitted in fiber. Based on the SPP excited on its surface and the highly bright sub-10 nm light spot formed by the SPP focusing at its apex, it was applied to achieve three sensing functions.

First, due to the dependence of the wavelength that satisfies the condition for SPP excitation on the RI of the external medium, the spiral-grating tapered gold tip was used as an SPR microsensor with a high space–time resolution. Based on such a microsensor, the diffusion processes of water–glycerol, water–sodium chloride and ethanol–water diffusion systems were monitored in situ, and the non-Fickian diffusion laws present in them were revealed. The monitoring of non-Fickian diffusion processes indicates that the practicability and advantage of the spiral-grating tapered gold tip microsensor in studying diffusion laws in liquids, and it is expected to be used to study the liquid diffusion behavior in batteries, organisms and other systems. In addition, the study of liquid–liquid diffusion by the microsensor also provides new ideas for the application of the rapidly developing optical fiber SPR sensors. They can not only be used for detection engineering in fields such as energy, medicine and chemistry, where previous applications of optical fiber SPR sensing were mainly focused, but also be utilized to advance research on fundamental scientific issues.

Second, due to the localized electromagnetic field caused by the excited SPP on the surface, the spiral-grating tapered gold tip was used as a PEF optical fiber sensor. It can achieve fluorescence detection with portable, user-friendly, and remote operation, may become a competitive device in compact fluorescence spectroscopy, and is expected to be applied in biotechnology, clinical analysis and analytical chemistry fields.

Third, based on the highly bright sub-10 nm light spot at the apex, the spiral-grating tapered gold tip served as a high-resolution, high-throughput, and high-contrast SNOM probe and was used to achieve sub-10 nm visualization of exciton and trion in MoSe_2_. This probe is expected to clearly measure and analyze the physical, chemical and biological characteristics of single molecules and other micro-nano substances by using the high bright sub-10 nm light spot formed at its apex as the illumination source. In addition, the probe can be combined with femtosecond pulsed laser to construct a microscopic imaging and spectral measurement system with high space–time resolution, which may become an ideal tool for in-depth study of the light–matter interaction at the micro-nano scale and exploring the interaction of multi-physics fields.

## Data Availability

No new data were created or analyzed in this study. Data sharing is not applicable.

## References

[1] Wang Q., Ren Z.H., Zhao W.M., Wang L., Yan X., Zhu A.S., Qiu F.M., Zhang K.K. (2022). Research advances on surface plasmon resonance biosensors. Nanoscale.

[2] Duan Q., Liu Y., Chang S., Chen H., Chen J.H. (2021). Surface plasmonic sensors: Sensing mechanism and recent applications. Sensors.

[3] dos Santos P.S.S., de Almeida J.M.M.M., Pastoriza-Santos I., Coelho L.C.C. (2021). Advances in Plasmonic Sensing at the NIR—A Review. Sensors.

[4] Steglich P., Lecci G., Mai A. (2022). Surface plasmon resonance (SPR) spectroscopy and photonic integrated circuit (PIC) biosensors: A comparative review. Sensors.

[5] Wang H., Wang T., Yuan X., Wang Y., Yue X., Wang L., Zhang J., Wang J. (2023). Plasmonic nanostructure biosensors: A review. Sensors.

[6] Xu Y., Bai P., Zhou X., Akimov Y., Png C.E., Ang L.K., Knoll W., Wu L. (2019). Optical refractive index sensors with plasmonic and photonic structures: Promising and inconvenient truth. Adv. Opt. Mater..

[7] Jing J., Liu K., Jiang J., Xu T., Wang S., Ma J., Zhang Z., Zhang W., Liu T. (2021). Performance improvement approaches for optical fiber SPR sensors and their sensing applications. Photon. Res..

[8] Gupta B.D., Kant R. (2018). Recent advances in surface plasmon resonance based fiber optic chemical and biosensors utilizing bulk and nanostructures. Opt. Laser Technol..

[9] Caucheteur C., Guo T., Albert J. (2015). Review of plasmonic fiber optic biochemical sensors: Improving the limit of detection. Anal. Bioanal. Chem..

[10] Zhao Y., Tong R.J., Xia F., Peng Y. (2019). Current status of optical fiber biosensor based on surface plasmon resonance. Biosens. Bioelectron..

[11] Liu Y., Peng W. (2021). Fiber-optic surface plasmon resonance sensors and biochemical applications: A review. J. Light. Technol..

[12] Cao S., Shao Y., Wang Y., Wu T., Zhang L., Huang Y., Zhang F., Liao C., He J., Wang Y. (2018). Highly sensitive surface plasmon resonance biosensor based on a low-index polymer optical fiber. Opt. Express.

[13] Li C., Gao J., Shafi M., Liu R., Zha Z., Feng D., Liu M., Du X., Yue W., Jiang S. (2021). Optical fiber SPR biosensor complying with a 3D composite hyperbolic metamaterial and a graphene film. Photon. Res..

[14] Soares M.S., Silva L.C.B., Vidal M., Loyez M., Facão M., Caucheteur C., Segatto M.E.V., Costa F.M., Leitão C., Pereira S.O. (2022). Label-free plasmonic immunosensor for cortisol detection in a D-shaped optical fiber. Biomed. Opt. Express.

[15] Wang Y., Rao X., Wu X., Chen G.Y., Liao C., Smietana M.J., Wang Y. (2024). Highly-Sensitive Polymer Optical Fiber SPR Sensor for Fast Immunoassay. Photonic Sens..

[16] Wang J., Lu X., Mi C., Yin Q., Lv J., Yang L., Liu W., Yi Z., Liu Q., Chu P.K. (2024). Ultra-high sensitivity photonic crystal fiber sensor based on dispersion turning point sensitization of surface plasmonic polariton modes for low RI liquid detection. Opt. Express.

[17] Lu X., Yu X., Zhou J., Chang M., Lu D. (2025). An ultra-wide range D-shaped Fiber SPR Sensor with a nanostructure of Gold–MoS_2_ and sodium for the simultaneous measurement of refractive index and temperature. Sensors.

[18] Parveen S., Pathak A., Gupta B.D. (2017). Fiber optic SPR nanosensor based on synergistic effects of CNT/Cu-nanoparticles composite for ultratrace sensing of nitrate. Sens. Actuators B Chem..

[19] Qian S., Zhang Y., Yuan H., Ji W., Liu Y., Zhao J., Han M., Peng W. (2018). Boronic acid functionalized fiber-optic SPR sensors for high sensitivity glycoprotein detection. Sens. Actuators B Chem..

[20] Jain S., Paliwal A., Gupta V., Tomar M. (2022). Smartphone integrated handheld Long Range Surface Plasmon Resonance based fiber-optic biosensor with tunable SiO_2_ sensing matrix. Biosens. Bioelectron..

[21] Gahlaut S.K., Pathak A., Gupta B.D., Singh J.P. (2022). Portable fiber-optic SPR platform for the detection of NS1-antigen for dengue diagnosis. Biosens. Bioelectron..

[22] Zheng W.L., Zhang Y.N., Li L.K., Li X.G., Zhao Y. (2022). A plug-and-play optical fiber SPR sensor for simultaneous measurement of glucose and cholesterol concentrations. Biosens. Bioelectron..

[23] Zheng W., Li Z., Kong W., Zhao Y., Zhang Y.N., Zhou E., Fan Y., Xu D., Gu T. (2025). Label-free detection of heme in electroactive microbes using a probe-typed optical fiber SPR sensor. Biosens. Bioelectron..

[24] Esteban Ó., Naranjo F.B., Díaz-Herrera N., Valdueza-Felip S., Navarrete M.C., González-Cano A. (2011). High-sensitive SPR sensing with Indium Nitride as a dielectric overlay of optical fibers. Sens. Actuators B Chem..

[25] Ding Z.W., Lang T.T., Wang Y., Zhao C.L. (2017). Surface plasmon resonance refractive index sensor based on tapered coreless optical fiber structure. J. Light. Technol..

[26] Peng Y., Zhao Y., Hu X.G., Yang Y. (2020). Optical fiber quantum biosensor based on surface plasmon polaritons for the label-free measurement of protein. Sens. Actuators B Chem..

[27] Liu Z., Liu W., Lai B., Zhang Y., Zhang Y., Yang X., Zhang J., Yuan L. (2021). SPR sensor based on Bessel-like beam. Opt. Express.

[28] Verma R.K., Gupta B.D. (2008). Theoretical modelling of a bi-dimensional U-shaped surface plasmon resonance based fibre optic sensor for sensitivity enhancement. J. Phys. D Appl. Phys..

[29] Li D., Yu S., Sun C., Zou C., Yu H., Xu K. (2015). U-shaped fiber-optic ATR sensor enhanced by silver nanoparticles for continuous glucose monitoring. Biosens. Bioelectron..

[30] Boruah B.S., Biswas R. (2018). Localized surface plasmon resonance based U-shaped optical fiber probe for the detection of Pb^2+^ in aqueous medium. Sens. Actuators B Chem..

[31] Arcas A.D.S., Dutra F.D.S., Allil R.C., Werneck M.M. (2018). Surface plasmon resonance and bending loss-based U-shaped plastic optical fiber biosensors. Sensors.

[32] Luo Z., Wang Y., Xu Y., Wang X., Huang Z., Chen J., Li Y., Duan Y. (2019). Ultrasensitive U-shaped fiber optic LSPR cytosensing for label-free and in situ evaluation of cell surface N-glycan expression. Sens. Actuators B Chem..

[33] Song H., Wang Q., Zhao W.M. (2020). A novel SPR sensor sensitivity-enhancing method for immunoassay by inserting MoS_2_ nanosheets between metal film and fiber. Opt. Laser Eng..

[34] Zhang H., Li X., Zhou X., Gong P., Zhao Y. (2023). U-fiber-based biosensor for temperature-compensated acetylcholine-specific measurement. Opt. Lett..

[35] Bombardi F.M., Muller M., Fabris J.L. (2024). U-shaped fiber sensor based on surface plasmon resonance of gold nanoparticles for measuring glyphosate in water. J. Light. Technol..

[36] Chang P., Zhang Y., Zhang A., Li Z., Wang Z., Shi Y. (2025). High-sensitivity U-shaped biosensor for rabbit IgG detection based on PDA/AuNPs/PDA sandwich structure. Nanophotonics.

[37] Li M., Singh R., Marques C., Zhang B., Kumar S. (2021). 2D material assisted SMF-MCF-MMF-SMF based LSPR sensor for creatinine detection. Opt. Express.

[38] Zhang J., Mai X., Hong X., Chen Y., Li X. (2022). Optical fiber SPR biosensor with a solid-phase enzymatic reaction device for glucose detection. Sens. Actuators B Chem..

[39] Caucheteur C., Guo T., Liu F., Guan B.O., Albert J. (2016). Ultrasensitive plasmonic sensing in air using optical fibre spectral combs. Nat. Commun..

[40] Liu F., Albert J. (2019). 40 GHz-rate all-optical cross-modulation of core-guided near infrared light in single mode fiber by surface plasmons on gold-coated tilted fiber Bragg gratings. APL Photon..

[41] Liu F., Zhang X., Li K., Guo T., Ianoul A., Albert J. (2021). Discrimination of bulk and surface refractive index change in plasmonic sensors with narrow bandwidth resonance combs. ACS Sens..

[42] Lobry M., Fasseaux H., Loyez M., Chah K., Goormaghtigh E., Wattiez R., Chiavaioli F., Caucheteur C. (2021). Plasmonic fiber grating biosensors demodulated through spectral envelopes intersection. J. Light. Technol..

[43] Ortega-Gomez A., Loyez M., Lobry M., Chah K., Zubia J., Villatoro J., Caucheteur C. (2021). Plasmonic sensors based on tilted Bragg gratings in multicore optical fibers. Opt. Express.

[44] Wang R., Zhang H., Liu Q., Liu F., Han X., Liu X., Li K., Xiao G., Albert J., Lu X. (2022). Operando monitoring of ion activities in aqueous batteries with plasmonic fiber-optic sensors. Nat. Commun..

[45] Liang Y., Zhang H., Zhu W., Agrawal A., Lezec H., Li L., Peng W., Zou Y., Lu Y., Xu T. (2017). Subradiant dipolar interactions in plasmonic nanoring resonator array for integrated label-free biosensing. ACS Sens..

[46] Consales M., Quero G., Spaziani S., Principe M., Micco A., Galdi V., Cutolo A., Cusano A. (2020). Metasurface-enhanced lab-on-fiber biosensors. Laser Photonics Rev..

[47] Kim H.M., Lee H.Y., Park J.H., Lee S.K. (2022). Fiber optic plasmonic sensors based on nanodome arrays with nanogaps. ACS Sens..

[48] Kong L.X., Zhang Y.X., Zhang W.G., Zhang Y.S., Yan T.Y., Geng P.C., Wang B. (2019). Lab-on-tip: Protruding-shaped all-fiber plasmonic microtip probe toward in-situ chem-bio detection. Sens. Actuators B Chem..

[49] Kong L.X., Chi M.J., Ren C., Ni L.F., Li Z., Zhang Y.S. (2022). Micro-lab on tip: High-performance dual-channel surface plasmon resonance sensor integrated on fiber-optic end facet. Sens. Actuators B Chem..

[50] Wang F., Li X., Wang S., Cao Y., Zhang L., Zhao Y., Dong X., Zheng M., Liu H., Lu W. (2023). 3D fiber-probe surface plasmon resonance microsensor towards small volume sensing. Sens. Actuators B Chem..

[51] Ge C., Peng Y., Shi J., Zhao Y. (2025). Miniature fiber end-integrated reflective surface plasmon resonance biosensor with chimney-like structure for ultra-sensitive alpha-fetoprotein detection. Sens. Actuators A Phys..

[52] Kawata S., Sun H.B., Tanaka T., Takada K. (2001). Finer features for functional microdevices. Nature.

[53] Jia B., Li J., Gu M. (2007). Two-photon polymerization for threedimensional photonic devices in polymers and nanocomposites. Aust. J. Chem..

[54] Huang R., Chen Y., Zhang J., Li Z.Y. (2025). In Situ Monitoring of Water–Sodium Chloride Diffusion via a Fiber-Integrated Plasmonic Microsensor. J. Phys. Chem. Lett..

[55] Li J., Mu J., Wang B., Ding W., Liu J., Guo H., Li W., Gu C., Li Z.Y. (2014). Direct laser writing of symmetry-broken spiral tapers for polarization-insensitive threedimensional plasmonic focusing. Laser Photonics Rev..

[56] Long L., Deng Q., Huang R., Li Z.Y. (2022). Plasmonic enhanced fluorescence via 3D printing spiral conical tapered gold tip bound to optical fiber. APL Photon..

[57] Long L., Deng Q., Huang R., Li J., Li Z.Y. (2023). 3D printing of plasmonic nanofocusing tip enabling high resolution, high throughput and high contrast optical near-field imaging. Light. Sci. Appl..

[58] Huang R., Long L., Deng Q., Li Z.Y. (2023). 3D printing of fiberintegrated plasmonic micro-grating tip enabling high-resolution realtime and in-site refractive index sensing. Opt. Laser Technol..

[59] Huang R., Long L., Yang H., Chen Y., Deng Q., Ju W., Li Z. (2024). In situ monitoring of non-Fickian liquid-liquid diffusion with a high space-time resolution fiber-integrated plasmonic sensor. ACS Photon..

[60] Cussler E.L. (2009). Diffusion: Mass Transfer in Fluid Systems.

[61] Fick A.V. (1855). On liquid diffusion. Lond. Edinb. Dublin. Philos. Mag. Sci..

[62] Williams J.W., Cady L.C. (1934). Molecular diffusion in solution. Chem. Rev..

[63] Vorobev A. (2014). Dissolution dynamics of miscible liquid/liquid interfaces. Curr. Opin. Colloid Interface Sci..

[64] Stokes R.H. (1950). An improved diaphragm-cell for diffusion studies, and some tests of the method. J. Am. Chem. Soc..

[65] Stokes R.H. (1951). Integral diffusion coefficients of potassium chloride solutions for calibration of diaphragm cells. J. Am. Chem. Soc..

[66] Lewis J.B. (1955). Some determinations of liquid-phase diffusion coefficients by means of an improved diaphragm cell. J. Appl. Chem..

[67] Mills R., Woolf L.A., Watts R.O. (1968). Simplified procedures for diaphragm—Cell diffusion studies. AIChE J..

[68] Choy E.M., Evans D.F., Cussler E.L. (1974). Selective membrane for transporting sodium ion against its concentration gradient. J. Am. Chem. Soc..

[69] Clunie J.C., Li N., Emerson M.T., Baird J.K. (1990). Theory and measurement of the concentration dependence of the differential diffusion coefficient using a diaphragm cell with compartments of unequal volume. J. Phys. Chem..

[70] Suhaimi H., Wang S., Das D.B. (2015). Glucose diffusivity in cell culture medium. Chem. Eng. J..

[71] Taylor G.I. (1953). Dispersion of soluble matter in solvent flowing slowly through a tube. Proc. R. Soc. A.

[72] Cottet H., Biron J.P., Martin M. (2007). Taylor dispersion analysis of mixtures. Anal. Chem..

[73] Cipelletti L., Biron J.P., Martin M., Cottet H. (2015). Measuring arbitrary diffusion coefficient distributions of nano-objects by taylor dispersion analysis. Anal. Chem..

[74] D’Errico G., Ortona O., Capuano F., Vitagliano V. (2004). Diffusion coefficients for the binary system glycerol + water at 25 °C. A velocity correlation study. J. Chem. Eng. Data.

[75] Culbertson C.T., Jacobson S.C., Ramsey J.M. (2002). Diffusion coefficient measurements in microfluidic devices. Talanta.

[76] Loureiro F.C., Neto A.B., Moreira C.S., Lima A.M., Neff H. (2011). A method for determining the mutual diffusion coefficient of molecular solutes based on surface plasmon resonance sensing. Sens. Actuators B Chem..

[77] Wiener O. (1893). Darstellung gekrummter Lichtstrahlen und Verwerthung derselben zur Untersuchung von Diffusion und Warmeleitung. Ann. Phys..

[78] Antrim D., Bunton P., Lewis L.L., Zoltowski B.D., Pojman J.A. (2005). Measuring the mutual diffusion coefficient for dodecyl acrylate in low molecular weight poly (dodecyl acrylate) with laser line deflection (Wiener’s method) and the fluorescence of pyrene. J. Phys. Chem. B.

[79] Petitjeans P., Maxworthy T. (1996). Miscible displacements in capillary tubes. Part 1. Experiments. J. Fluid Mech..

[80] Ambrosini D., Rastogi P.K. (2008). Diffusion measurements by optical methods: Recent advances and applications. Opt. Lasers Eng..

[81] Viner G., Pojman J.A. (2008). Studying diffusion of partially miscible and systems near their consolute point by laser line deflection. Opt. Lasers Eng..

[82] Lewis L.L., Massey K.N., Meyer E.R., McPherson J.R., Hanna J.S. (2008). New insight into isothermal frontal polymerization models: Wiener’s method to determine the diffusion coefficients for high molecular-weight poly (methyl methacrylate) with neat methyl methacrylate. Opt. Lasers Eng..

[83] Swapna M.N.S., Anitha M.J., Sankararaman S.I. (2017). Study of drug diffusion rate by laser beam deflection technique. J. Biomed. Opt..

[84] He M.G., Zhang S., Zhang Y., Peng S.G. (2015). Development of measuring diffusion coefficients by digital holographic interferometry in transparent liquid mixtures. Opt. Express.

[85] Kegeles G., Gosting L.J. (1947). The theory of an interference method for the study of diffusion. J. Am. Chem. Soc..

[86] Vitagliano V., Lyons P.A. (1956). Diffusion coefficients for aqueous solutions of sodium chloride and barium chloride. J. Am. Chem. Soc..

[87] Fernández-Sempere J., Ruiz-Beviá F., Colom-Valiente J., Más-Pérez F. (1996). Determination of diffusion coefficients of glycols. J. Chem. Eng. Data.

[88] Ternström G., Sjöstrand A., Aly G., Jernqvist Å. (1996). Mutual diffusion coefficients of water+ ethylene glycol and water + glycerol mixtures. J. Chem. Eng. Data.

[89] Chhaniwal V.K., Anand A., Girhe S., Patil D., Subrahmanyam N., Narayanamurthy C.S. (2003). New optical techniques for diffusion studies in transparent liquid solutions. J. Opt. A Pure Appl. Opt..

[90] Zhao C., Li J., Ma P. (2006). Diffusion studies in liquids by holographic interferometry. Opt. Laser Technol..

[91] Axelsson A., Marucci M. (2008). The use of holographic interferometry and electron speckle pattern interferometry for diffusion measurement in biochemical and pharmaceutical engineering applications. Opt. Lasers Eng..

[92] Torres J.F., Komiya A., Shoji E., Okajima J., Maruyama S. (2012). Development of phase-shifting interferometry for measurement of isothermal diffusion coefficients in binary solutions. Opt. Lasers Eng..

[93] Šeta B., Gavalda J., Bou-Ali M.M., Ruiz X., Santamaria C. (2020). Determining diffusion, thermodiffusion and Soret coefficients by the thermogravitational technique in binary mixtures with optical digital interferometry analysis. Int. J. Heat Mass Transf..

[94] Wu Q., Chen L., Komiya A. (2021). Dynamic imaging and analysis of transient mass transfer process using pixelated-array masked phase-shifting interferometry. Int. J. Heat Mass Transf..

[95] Sun L., Pu X. (2016). A novel visualization technique for measuring liquid diffusion coefficient based on asymmetric liquid-core cylindrical lens. Sci. Rep..

[96] Meng W., Xia Y., Song F., Pu X. (2017). Double liquid-core cylindrical lens utilized to measure liquid diffusion coefficient. Opt. Express.

[97] Wei L., Meng W., Chen Y., He B., Zhou Q., Pu X. (2020). Optical measurement of concentration-dependent diffusion coefficients of binary solution using an asymmetric liquid-core cylindrical lens. Opt. Lasers Eng..

[98] Wang R., Meng W., Zhang Y., Li D., Pu X. (2022). An improved method for measuring the concentration dependence of Fick diffusion coefficient based on Boltzmann equation and cylindrical liquid-core lenses. Int. Commun. Heat Mass Transf..

[99] Bouchaudy A., Loussert C., Salmon J.B. (2018). Steady microfluidic measurements of mutual diffusion coefficients of liquid binary mixtures. AIChE J..

[100] Nguyen H.T., Bouchaudy A., Salmon J.B. (2022). Microfluidic free interface diffusion: Measurement of diffusion coefficients and evidence of interfacial-driven transport phenomena. Phys. Fluids.

[101] Hamada M., de Anna P. (2023). A method to measure the diffusion coefficient in liquids. Transp. Porous Media.

[102] Ismagilov R.F., Stroock A.D., Kenis P.J., Whitesides G., Stone H.A. (2000). Experimental and theoretical scaling laws for transverse diffusive broadening in two-phase laminar flows in microchannels. Appl. Phys. Lett..

[103] Kirner T., Jaschinsky P., Köhler J.M. (2004). Spatially resolved detection of miniaturized reaction–diffusion experiments in chip reactors for educational purposes. Chem. Eng. J..

[104] Salmon J.B., Ajdari A., Tabeling P., Servant L., Talaga D., Joanicot M. (2005). In situ Raman imaging of interdiffusion in a microchannel. Appl. Phys. Lett..

[105] Dambrine J., Géraud B., Salmon J.B. (2009). Interdiffusion of liquids of different viscosities in a microchannel. New J. Phys..

[106] Broboana D., Balan C.M., Wohland T., Balan C. (2011). Investigations of the unsteady diffusion process in microchannels. Chem. Eng. Sci..

[107] Peters C., Wolff L., Haase S., Thien J., Brands T., Koß H.J., Bardow A. (2017). Multicomponent diffusion coefficients from microfluidics using Raman microspectroscopy. Lab Chip.

[108] Yamashita H., Kakuta N., Kawashima D., Yamada Y. (2018). Measurement of temperature-dependent diffusion coefficients of aqueous solutions by near-infrared simultaneous imaging of temperature and concentration. Biomed. Phys. Eng. Express.

[109] Iiyama T., Furuya M., Arai T. (2022). Near-infrared imaging to quantify the diffusion coefficient of sodium pentaborate aqueous solution in a microchannel. Chem. Eng. Sci..

[110] Huang R., Chen Y., Zhang J., Li Z.Y. (2026). Exotic Ethanol–Water Diffusion Mediated by Molecular Self-Assembly. J. Phys. Chem. Lett..

[111] Latifi H., Zibaii M.I., Hosseini S.M., Jorge P. (2012). Nonadiabatic tapered optical fiber for biosensor applications. Photon. Sens..

[112] Liu J., Xing Y., Zhou X., Chen G.Y., Shi H. (2021). Light-sheet skew rays enhanced U-shaped fiber-optic fluorescent immunosensor for Microcystin-LR. Biosens. Bioelectron..

[113] Mivelle M., van Zanten T.S., Neumann L., van Hulst N.F., Garcia-Parajo M.F. (2012). Ultrabright bowtie nanoaperture antenna probes studied by single molecule fluorescence. Nano Lett..

[114] Chen Y., Huang R., Zhang J., Chen J., Li Z.Y. (2025). Sub-10 nm Visualization of Trions in Ultralow-Strained Monolayer MoSe_2_. Nano Lett..

